# Regional Peer Effects of Corporate Tax Avoidance

**DOI:** 10.3389/fpsyg.2021.744371

**Published:** 2021-12-07

**Authors:** Ya Gao, Chang Cai, Yiwei Cai

**Affiliations:** ^1^School of Public Finance and Taxation, Central University of Finance and Economics, Beijing, China; ^2^Department of Computer Science, Data Science and Statistics, Faculty of Science, University of Bath, Bath, United Kingdom

**Keywords:** tax avoidance, peer effect, learning, communication, region

## Abstract

This study empirically demonstrates significant regional peer effects due to tax avoidance. We used peer companies’ idiosyncratic stock returns as an instrumental variable to address potential endogeneity problems. The heterogeneity analysis indicates that for companies with a stronger intensity of regional tax collection and management, a higher degree of informatization, and companies with a low management shareholding ratio, the regional peer effects of enterprise tax avoidance are more significant. Finally, we determined that the managers’ information learning, reputation consideration, and information communication are key mechanisms propagating peer effects. The conclusions of this paper enrich and expand the peer effect theory of corporate tax avoidance, thereby providing a theoretical basis and empirical evidence for tax authorities in supervising corporate tax avoidance.

## Introduction

Peer effects have received extensive attention in various fields including accounting, economics, and finance. Albuquerque (2009); Reppenhagen (2010), and Tse and Tucker (2010) found that managers’ disclosure decisions, accounting methods, and chief executive officers’ (CEOs’) relative performance assessments were subject to peer effects. The research of Gleason et al. (2008) and Beatty et al. (2013) determined that false financial reports distort peer companies’ financial reporting decisions and investment behavior. Gaviria and Raphael (2001); Mas and Moretti (2009), and Bandiera et al. (2010) asserted that there were peer effects in employees’ work efficiency and negative behaviors in teenagers, such as drug abuse, drinking, smoking, dropping out of school, and other activities. Others suggested that there are significant peer effects in a company’s investment behaviors, financing decision-making, illegal behaviors, and stock divisions (Leary and Roberts, 2014; Dougal et al., 2015; Kaustia and Rantala, 2015; Fracassi, 2017; Parsons et al., 2018). Lu et al. (2017) found that the capital structure of Chinese enterprises is affected by the capital structure of their peers. Further, they found that the influence mechanism of peer effects mainly includes the managers’ reputation consideration. Adhikari and Agrawal (2018) found that companies’ dividend and share repurchasing policies are significantly influenced by peer company policies. Grennan (2019) also confirmed that a company’s dividend distribution policy has a significant peer effect. While there is substantial literature documenting peer effects in other settings, the study of peer effects in taxes is novel; little research exists on peer effects in corporate tax avoidance and their impact mechanism. In this study, we investigate the role of peer effects in corporate tax avoidance.

Corporate tax avoidance is a global problem. Tax avoidance causes significant tax losses and reduces taxation’s ability to play its due role in serving national governance. Therefore, corporate tax avoidance is a hot topic in practice among government regulatory authorities and academia. However, the current research on corporate tax avoidance is primarily based on the assumption that companies make decisions independently. Studies describe factors that affect corporate tax avoidance regarding micro-level influencing factors, such as the operational characteristics of companies (Porcano, 1986; Derashid and Zhang, 2003) and the personal characteristics of executives (Dyreng et al., 2010; Chyz, 2013), and macro-level factors, such as financial development, regional marketization level, government governance level, and other institutional and environmental factors (Wingender, 2008; Cai and Liu, 2009; Ma and Li, 2012). Companies’ industry characteristics (Derashid and Zhang, 2003) and the market position of products (Brown et al., 2014; Kubick et al., 2015) are important factors affecting corporate tax avoidance. However, the aforementioned studies on corporate tax avoidance have ignored the interaction between corporate behavioral decisions and how companies’ peers affect corporate tax avoidance behavior. This study considers the interaction between companies and examines whether their peers affect the companies’ tax avoidance behavior.

This study selected A-share listed companies from 2008 to 2018 as the research object. First, it empirically tested the regional peer effects of tax avoidance among companies and found that significant regional peer effects exist. Second, for companies with a stronger intensity of regional tax collection and management, a higher degree of informatization, and companies with a low management shareholding ratio, the regional peer effects of enterprise tax avoidance are more significant. Third, in the mechanism analysis, this study found that the peer effects of corporate tax avoidance include information learning, reputation consideration, and information communication of managers. Peer effects are produced when smaller companies imitate and learn from larger companies or when younger companies imitate and learn from older companies. The shorter a CEO’s tenure, the younger their age, and the higher their education level, the more significant the peer effects of the listed companies’ tax avoidance. Additionally, when company managers have similar native places, ages, and educational backgrounds, the more significant the regional peer effects of tax avoidance are.

This study makes the following contributions. First, the social interaction between geographically adjacent enterprises is introduced into the tax behavior of enterprises. In addition, regional peer effects are analyzed to reveal the mutual imitation of tax avoidance decisions among enterprises, which enriches research on corporate tax motives, fills gaps in the related research fields, and provides new ideas for tax authorities to perform anti-tax avoidance work. Second, in the related literature on peer effects, most of a company’s investment decisions, violations, capital structure, dividend distributions, and other aspects are studied. This paper supplements and expands the literature on peer effects from the perspective of corporate tax avoidance. Third, this paper analyzes the mechanisms of corporate tax avoidance peer effects. Additionally, the local information, information learning, reputation consideration, and information communication of managers in corporate taxes are discussed in terms of the peer effects mechanism and influencing factors from the strength of local organs of tax collection and administration, which enriches the study of peer effects.

The remainder of the paper is organized as follows. The second part presents the related literature and the hypothesis development; the third part details the methodology; the fourth part presents the main empirical results, robustness tests, heterogeneity analysis, and the mechanism analysis; the fifth part is the conclusion.

## Related Literature and Hypotheses Development

### Existence of Peer Effects in Corporate Tax Avoidance

At present, many studies are pointing out the existence of peer effects in various behaviors of enterprises from different perspectives. Peer effects applied to the analysis of corporate tax avoidance can be summarized as follows.

First, managers sometimes avoid negative reputations by imitating the behavior of others (Scharfstein and Stein, 1990). When an enterprise’s tax avoidance behavior is more aggressive than its competitors, it is more likely to attract the attention of tax auditing departments and the public, thus increasing its tax inspection risk and reputational costs (Boning et al., 2018). Therefore, companies will adopt tax avoidance behaviors similar to their peers to reduce additional tax inspection risk and reputational costs.

Second, managers are very concerned about their reputations and competence information asymmetry (Zwiebel, 1995). In the managerial labor market, because of the competence information asymmetry, labor usually cannot accurately understand managers’ competence level, which is evaluated by observing their relative performance. Therefore, to ensure their performance and external reputation and gain recognition and favor from the demand side of labor, managers tend to adopt behavioral decisions similar to those at other enterprises. Because China’s capital market still requires improvement, information is not transparent, so a company’s controller cannot efficiently obtain valuable information to make decisions regarding the company’s future development. To balance the uncertainty between risks and benefits, managers pay attention to the decisions of their peers and obtain additional valuable information through peer behaviors to reduce the uncertainty risk as well as criticism and questioning by stakeholders.

Third, tax laws and regulations are complex and changeable. To control the tax burden and save tax planning costs, enterprises learn new tax avoidance strategies or infer acceptable tax planning strategies by observing their competitors (Brown and Drake, 2014). For example, enterprises can learn and imitate the tax avoidance behaviors of peer companies in various ways, transfer their business to areas with tax preferences, and reduce their tax burden by transferring it to affiliates. Therefore, we propose our first hypothesis, as follows.

**Hypothesis 1a:** Corporate tax avoidance has regional peer effects; that is, the tax avoidance behavior of enterprises in the same region significantly increases a company’s degree of tax avoidance.

In addition, spatial distance becomes unavoidable when considering the regional peer effects of corporate tax avoidance. From the perspective of classical spatial economics, the knowledge spillover effect holds that the closer the geographical location of the actors, the more convenient it is for them to meet, communicate frequently, and establish a trust relationship, thus promoting the transmission of knowledge and the spillover effect (Filippi and Torre, 2003). Therefore, enterprises in the same region are more likely to learn from and imitate each other because of their close geographical distances and similar institutional environments. This imitation behavior will gradually weaken as distance increases between the two enterprises. Based on this analysis, this paper proposes the following hypothesis.

**Hypothesis 1b:** Corporate tax avoidance is positively influenced by companies with a similar geographical distance. This influence gradually decreases as the geographical distance increases between the two companies.

### Heterogeneity Analysis of Regional Peer Effects in Corporate Tax Avoidance

Improving the intensity of regional tax collection and management can deter the tax avoidance behavior of taxpayers and reduce the degree of tax avoidance. However, when enterprises face stronger tax collection and management intensity, they imitate peer enterprises’ tax avoidance decisions to ensure the legitimacy of their tax avoidance decisions. As such, this paper puts forward the following hypothesis.

**Hypothesis 2a:** The degree of tax collection and management in the region enhances the regional peer effects of corporate tax avoidance.

The degree of informatization in the region where an enterprise is located will affect its information acquisition and communications. Generally speaking, when a region has a high degree of informatization, the cost of obtaining information between enterprises is relatively low, communication is more convenient, and peer effects are more likely to occur. Thus, this paper proposes the following hypothesis.

**Hypothesis 2b:** The higher the degree of regional informatization, the more pronounced the regional peer effects of corporate tax avoidance.

Based on the principal–agent theory, scholars believe that the separation of ownership and management rights enables enterprise managements to accept the entrustment of enterprise owners to perform production and operation activities within the scope of a contract signed in advance. However, because the interests of the trustee, as the enterprise management, and the principal, as the enterprise owner, are often inconsistent, the principal–agent problem occurs. To maximize their interests, the management of the enterprise usually exhibits opportunistic behavior, which damages the interests of the enterprise owner. The principal–agent problem has also been applied to corporate tax avoidance by scholars who put tax avoidance into the principal–agent framework and believe that corporate tax avoidance is mostly caused by the self-interested behavior of corporate executives.

The existing literature shows that management ownership can make managers’ behavior and shareholders’ interests consistent, and with an increase in management shareholding, the management’s interests and the shareholders’ interests become more consistent, which can alleviate the principal–agent problem. In other words, when the management ownership is at the level at which the management’s interests are consistent with the shareholders’ interests, managers are likely to engage in activities to maximize corporate performance (Morck et al., 1988). When the management ownership is at a level at which the management’s interests are consistent with the shareholders’ interests, the management’s self-interest motive will decrease to imitate peer enterprises’ tax avoidance behaviors. Therefore, this paper proposes the following hypothesis.

**Hypothesis 2c:** The lower the shareholding ratio of management, the more inconsistent the interests of management and shareholders and the more significant the regional peer effect of corporate tax avoidance and vice versa.

### Mechanism Analysis of Regional Peer Effects in Corporate Tax Avoidance

After revealing the regional peer effects of corporate tax avoidance, it is important to suggest how to govern the peer effects of tax avoidance effectively. Therefore, it is essential to explore the occurrence mechanism of peer effects. On one hand, the behavioral decisions of leading companies often have significant guiding value; therefore, companies will consciously imitate and refer to the tax avoidance decisions of leading companies in the region. On the other hand, a social network is conducive to information transmission between individuals and enterprises. Similarly, as social individuals, a social network of company managers will also affect behavioral decisions. Therefore, this paper focuses on three perspectives: manager information learning, manager reputation consideration, and manager information communication.

Concerning manager information learning, larger enterprises with more capital and political resources can obtain more tax information and develop more professional tax planning strategies (Porcano, 1986). Therefore, enterprises will imitate the tax behavior of leading or large enterprises (Xiao, 2021) and form tax avoidance strategies to improve the legitimacy and credibility of their behavior and avoid being questioned and criticized by other stakeholders in an uncertain environment (Franco et al., 2019). Therefore, this paper puts forward the following hypothesis.

**Hypothesis 3a:** The formation of corporate tax avoidance peer effects partly comes from the tendency for smaller and younger enterprises to imitate and learn the tax avoidance behaviors of larger and older enterprises.

Manager reputation consideration shows that to ensure their performance and external reputation and gain recognition and favor from the demand side of labor, managers tend to adopt behavior similar to managers from other enterprises. Therefore, this paper puts forward the following hypothesis.

**Hypothesis 3b:** The more important reputation is to a manager, the greater the incentive to maintain similar tax avoidance decisions to those of peer firms.

Manager information communication shows that executives in companies in the same region usually live in the same area. Because of their close geographical distance, it is convenient to establish social networks and communicate and learn frequently, thus lowering the cost of information collection (Ayers et al., 2011). Therefore, this paper proposes the following hypothesis.

**Hypothesis 3c:** The regional peer effects of corporate tax avoidance stems in part from communication among executives.

## Methodology and Data

### Data Source

The financial data in this paper come from the China Stock Market and Accounting Research Database. The nominal income tax rate data are derived from the notes and information of the financial statements provided by the WIND database; this is the nominal tax rate of listed companies that enjoy preferential treatment.

The sample starts in 2008 because of the concerns that China implemented a new company income tax law in 2008. The sample comprised all the companies listed in the China A-shares from 2008 to 2018 to ensure the consistency of the tax environment of companies. We define the province, autonomous region, and municipality directly under the central government in which the listed company’s headquarters is located as “the same region,” and there are 31 regions. The following data were processed in this paper: those for which (1) the total profit is less than or equal to zero; the profit before interest and tax are less than or equal to zero; the income tax and tax are deleted. (2) The samples of ST (special treatment) stocks and the financial insurance industry were excluded, and samples with an effective income tax rate less than zero and greater than one were eliminated. (3) Samples with abnormal and missing key variables were eliminated. (4) Fewer than five companies in the industry were excluded.

### Variable Definition

#### Tax Avoidance Degree of the Company

According to Desai and Dharmapala (2006), the book-tax difference (BTD) and the difference between nominal income tax rate and effective income tax rate are used to measure the tax avoidance degree of the company.

For the first tax avoidance index, to calculate the BTD, we first present the following definition of taxable income:


(1) $$\mathrm{taxable}\mathrm{income}=\frac{\mathrm{income}\mathrm{tax}\mathrm{expense}-\mathrm{deferred}\mathrm{income}\mathrm{tax}\mathrm{expense}}{\mathrm{nominal}\mathrm{income}\mathrm{tax}\mathrm{rate}}$$

(2) $$\mathrm{BTD}=\frac{\mathrm{pre}\text{-}\mathrm{tax}\mathrm{accounting}\mathrm{profit}-\mathrm{taxable}\mathrm{income}}{\mathrm{Total}\mathrm{Assets}}$$

The greater the BTD, the stronger the tax avoidance motivation.

The nominal income tax rate data is derived from the notes in-formation of financial statements provided by the WIND database. This tax rate is the nominal tax rate after the listed companies enjoy preferential treatment. Thus,


(3) $$\mathrm{ERT}=\frac{\mathrm{income}\mathrm{tax}}{\mathrm{Earnings}\mathrm{Before}\mathrm{Interest}\mathrm{and}\mathrm{Tax}}$$

the nominal income tax rate data is derived from the notes information of financial statements provided by the WIND database. This tax rate is the nominal tax rate after the listed companies enjoy preferential treatment. Thus,


(4) DR=nominaltaxrate-ERT

The higher the DR, the higher the tax avoidance degree of the companies.

#### Peer Influence

The key explanatory variable in this study is tax avoidance peer influence. We used the same region to define peer groups. The peer enterprises’ degree of tax avoidance is represented by the arithmetic mean of the degree of tax avoidance of other enterprises in the same region, excluding the enterprises themselves. Thus, the tax avoidance degree of the peer companies (PBTD and PDR) adopts the average of all firms (excluding firm *i*).

#### Control Variables

The other variables in this study include firm-specific and peer firm average characteristics. The firm-specific characteristics include the variables constructed as firm *i*’s value in year *t*. Specifically, these include the following. (1) *SIZE* is the company’s scale, calculated as the logarithm of total assets; (2) *ROA* captures profitability, calculated as the ratio of net income before interest on debt to total assets; (3) *LEV* captures leverage, calculated as the ratio of total liabilities on debt to total assets; (4) *ROI* is the company’s return on investment, calculated as the ratio of the income from investment on debt to total assets; (5) *PPE* is the company’s tangible asset intensity, calculated as the net value of fixed assets on debt to total assets; (6) *INVENT* is the company’s inventory intensity, calculated as the net value of the inventory on debt to total assets; (7) *INTANG* is the company’s intangible asset intensity, calculated as the net value of the intangible assets on debt to total assets; and (8) *AGE* is logarithmic with the age of the company, defined as the period beginning with the company’s listing on the stock exchange. The peer firm’s characteristics are represented by the arithmetic mean of the degree of the aforementioned firm-specific covariates of other enterprises in the same region except for the enterprises themselves. We denote them as *PSIZE*, *PROA*, *PLEV*, *PROI*, *PPPE*, *PINVENT*, *PINTANG*, and *PAGE*. Table 1 provides the summary statistics for the main variables used in the regressions.

**TABLE 1:** Summary statistics.

Variable name	Sample size	Mean	SD	Min	Max
**Dependent variables**					
BTD	12,561	−0.01	0.10	−0.54	8.56
DR	12,561	−0.01	0.12	−0.62	0.24
**Independent variables**					
PBTD	12,561	−0.01	0.02	−0.08	0.12
PDR	12,561	−0.01	0.02	−0.22	0.08
**Firm-specific covariates**					
SIZE	12,561	21.96	1.28	15.72	28.51
ROA	12,561	0.06	0.12	0.00	11.01
LEV	12,561	0.43	0.27	0.01	18.14
ROI	12,561	0.01	0.02	−0.08	0.52
PPE	12,561	0.23	0.17	0.00	0.93
INVENT	12,561	0.17	0.16	0.00	0.94
INTANG	12,561	0.05	0.06	0.00	0.85
AGE	12,561	2.70	0.35	0.00	4.08
**Peer firm average covariates**					
PSIZE	12,561	21.89	0.37	20.79	26.11
PROA	12,561	0.07	0.02	0.02	0.19
PLEV	12,561	0.43	0.06	0.30	1.14
PROI	12,561	0.01	0.00	−0.00	0.04
PPPE	12,561	0.22	0.05	0.08	0.49
PINVENT	12,561	0.16	0.03	0.04	0.35
PINTANG	12,561	0.05	0.01	0.01	0.13
PAGE	12,561	2.68	0.15	2.14	3.23
**Instrumented instrument**					
IV	11,610	−0.01	0.03	−0.09	0.09

### Empirical Strategy

#### Empirical Model

This paper’s empirical analysis aims to prove the existence and influencing mechanisms of regional peer effects in corporate tax avoidance. This study draws on the method used by Dougal et al. (2015) to create a benchmark regression model, is as follows:


(5) $$Tax_{ist}=\beta_{0}+\beta_{1}\cdot Peer_{-ist}+\beta_{2}\cdot X_{ist}+\beta_{3}\cdot Z_{-ist}+\rho_{i}+\delta_{t}+\varepsilon_{ist}$$

where the indices *i*, *s*, and *t* correspond to firm, region, and year, respectively. The independent variable, *Tax*_*ist*_, is the tax avoidance degree of firm *i*. The covariate *Peer_–*ist*_* denotes peer companies’ average outcome in region *s* (excluding firm *i*). *X*_*ist*_ contains peer firm average characteristics. *Z_–*ist*_* is a control variable that changes over time at the regional level and represents the average tax avoidance influencing factors of the peer enterprises of firm *i* in region *s* (excluding the sample of companies in the same industry as company *i*, in the region, the same below). Firm and year fixed effects are represented by ρ_*i*_ and δ_*t*_, respectively. Finally, ε is the unobservable error component. Below, the coefficient estimates are the test statistics from robust standard errors clustered by the industry. We are mainly concerned about the estimation results of coefficient β_1_. If the tax avoidance behavior of peers directly affects the tax avoidance behavior of companies, β_1_ is significant, indicating the peer effects of tax avoidance.

#### Endogeneity and Instrumental Variable

As Manski (1993) put forth, the “reflection problem” and inevitable omitted variables cause many difficulties in identifying peer effects. Therefore, we used regional peer companies’ (excluding the sample of companies in the same industry as the company *i* in the region) idiosyncratic returns as an instrumental variable to identify peer effects, a design pioneered by Leary and Roberts (2014). The instrumental variable has the following characteristics:

First, it is related to the endogenous explanatory variable. A change in a company’s future expected cash flow affects idiosyncratic stock returns (Campbell et al., 2001). The higher the idiosyncratic stock returns, the more the expected cash flow, the higher the expected tax burden, and the higher the motivation for tax avoidance. Therefore, there is a positive correlation between tax avoidance and idiosyncratic stock returns.^1^ Second, it is exogenous. A company’s idiosyncratic returns only contain information about the company itself, so the idiosyncratic returns of peer companies can only affect the tax avoidance behavior of peer companies and cannot directly affect the tax avoidance behavior of the firm *i*. Thus, the instrumental variable used in this paper is effective.

## Empirical Results

### Model

The results of the baseline model are shown in Table 2. The results indicate that obvious tax avoidance peer effects exist in listed companies in China, and peers significantly affect companies’ tax avoidance behavior. The result is consistent with Hypothesis 1a. Specifically, columns (1)–(3) and columns (5)–(7) are the ordinary least squares (OLS) regression results with *BTD* and *DR* as the dependent variables when the firm, year, and region fixed effects are included. Columns (2) and (6) include firm-specific covariates to control for other market frictions that could drive changes in the degree of tax avoidance. Columns (3) and (7) include firm- and region-specific covariates to control for other market frictions and regional dynamics that could cause changes in the degree of tax avoidance.

**TABLE 2:** Existence of regional peer effects in corporate tax avoidance.

	BTD				DR			
	OLS			2SLS	OLS			2SLS
	(1)	(2)	(3)	(4)	(5)	(6)	(7)	(8)
PBTD	0.048** (0.023)	0.051** (0.023)	0.076** (0.032)	0.991** (0.447)				
PDR					0.125** (0.054)	0.127** (0.061)	0.143*** (0.055)	1.311* (0.722)
SIZE		0.007*** (0.002)	0.007*** (0.001)	0.008*** (0.002)		0.010 (0.006)	0.008** (0.003)	0.006 (0.005)
ROA		0.160*** (0.018)	0.161*** (0.010)	0.038** (0.016)		0.565*** (0.047)	0.063*** (0.009)	0.084 (0.055)
LEV		−0.024*** (0.005)	−0.024*** (0.004)	−0.032*** (0.007)		−0.071*** (0.016)	−0.090*** (0.012)	−0.096*** (0.018)
ROI		0.199*** (0.049)	0.200*** (0.027)	0.265*** (0.053)		0.341** (0.153)	0.564*** (0.084)	0.564*** (0.135)
PPE		−0.028*** (0.006)	−0.027*** (0.005)	−0.032*** (0.008)		−0.011 (0.024)	−0.025* (0.014)	−0.034 (0.022)
INVENT		−0.022*** (0.008)	−0.022*** (0.006)	−0.018* (0.010)		−0.024 (0.022)	−0.028 (0.018)	−0.019 (0.027)
INTANG		−0.004 (0.019)	−0.004 (0.013)	−0.004 (0.025)		−0.026 (0.046)	−0.040 (0.040)	−0.022 (0.056)
AGE		−0.016** (0.008)	−0.014** (0.007)	−0.019 (0.012)		0.028 (0.029)	−0.003 (0.018)	0.011 (0.032)
PSIZE			−0.000 (0.003)	−0.009 (0.006)			−0.019** (0.008)	0.007 (0.016)
PROA			−0.024 (0.032)	−0.658** (0.318)			0.073 (0.072)	−0.182 (0.198)
PLEV			−0.016** (0.008)	0.005 (0.020)			0.056* (0.032)	0.039 (0.047)
PROI			−0.050 (0.123)	−0.235 (0.190)			0.191 (0.336)	−0.981 (0.847)
PPPE			0.050** (0.025)	−0.029 (0.050)			−0.025 (0.060)	0.001 (0.105)
PINVENT			0.056** (0.026)	0.033 (0.043)			−0.131* (0.071)	0.142 (0.184)
PINTANG			−0.021 (0.055)	0.052 (0.078)			−0.046 (0.162)	0.235 (0.231)
PAGE			−0.017* (0.010)	0.001 (0.019)			−0.055** (0.024)	0.031 (0.052)
Firm\year fixed effects	Yes	Yes	Yes	Yes	Yes	Yes	Yes	Yes
Cons	−0.015*** (0.000)	−0.106*** (0.039)	−0.071 (0.070)		−0.010*** (0.001)	−0.301** (0.121)	0.434*** (0.158)	
*N*	12,333	12,328	12,328	11,409	12,333	12,328	12,328	11,409
*R* ^2^	0.710	0.726	0.726	–	0.447	0.475	0.454	–
First-stage results								
				0.023*** (0.003)				0.043*** (0.005)
*K-P F*	–	–	–	51.084	–	–	–	87.512

*Robust standard errors clustered at the industry level are presented in parentheses. K-P F value is Kleibergen–Paap F value.*

The symbols ***, **, and * denote significance at the 0.01, 0.05, and 0.1 levels, respectively.

Columns (4) and (8) are the two-stage least squares (2SLS) estimation results, including firm- and region-specific covariates and firm and year-fixed effects. Their magnitudes are greater than their OLS counterparts. One possibility is that the endogeneity of *BTD* and *DR* causes a downward bias in the OLS estimation.

### Robustness Checks

To further verify the robustness of the benchmark model, this section presents robustness tests conducted from the aspects

of adjusting the measurement of tax avoidance degree and changing the method of identifying peer companies. We also used a dynamic panel data model to alleviate the endogeneity problem in this paper.

#### Adjusting the Measurement Method of Tax Avoidance Degree

In the robustness test, we used Hanlon and Heitzman’s (2010) methods and measured the tax avoidance degree of a company

using its effective tax rate. The definition of taxable income is as follows:


(6) $$\mathrm{ERT1}=\frac{\mathrm{income}\mathrm{tax}}{\mathrm{Earnings}\mathrm{Before}\mathrm{Interest}\mathrm{and}\mathrm{Tax}}$$

(7) $$\mathrm{ERT2}=\frac{\mathrm{income}\mathrm{tax}\mathrm{expense}-\text{deferred}\mathrm{income}\mathrm{tax}\mathrm{expense}}{\mathrm{Earnings}\mathrm{Before}\mathrm{Interest}\mathrm{and}\mathrm{Tax}}$$

The corresponding explanatory variables are *PERT1* and *PERT2*. The results are shown in Supplementary Appendix Table 1. In all cases, the tax avoidance peer effects are significant, and the magnitudes are very robust.

#### Changing the Identification Method of Peer Companies

In this paper’s benchmark regression, other enterprises in the same region (i.e., province, autonomous region, and municipality directly under the central government) other than the enterprise itself are adopted as the enterprise’s peer companies. To further verify the regional peer effects in tax avoidance and the influence of the geographical distance between enterprises on the degree of corporate tax avoidance, we take the corresponding company as the dot, the radius of different kilometers as the radius, and other enterprises within the radius of kilometers as the peer companies of this enterprise.

The specific approach is as follows. (1) Use Baidu Map application programming interface (API) to convert the office addresses of all A-share listed companies in China into the corresponding latitude and longitude data. (2) Calculate the distance between companies using the Haversine formula,^2^ according to longitude and latitude data. (3) Calculate the average tax avoidance degree of the peer enterprises.^3^ The regression results are shown in Supplementary Appendix Table 2. With the increase of geographical radius, the peer effects of corporate tax avoidance gradually weaken or even disappear, which verifies Hypothesis 1b.

#### Dynamic Panel Data Model

In the baseline regression, we used instrumental variables to alleviate the endogeneity problems in the model. To further alleviate the endogeneity problem of the explanatory variables, a system generalized method of moments estimation was used to estimate the parameters of the equation. Firstly, the GMM estimation of the two-step system tends to lead to a serious underestimate of the standard error of the parameter estimation when the sample is small (Windmeijer, 2005). To obtain an unbiased estimate of the standard error, we decided to adopt the method proposed by Windmeijer (2005) to revise the estimate of the standard error. Secondly, in the setting of instrumental variables, for the sake of robustness, we treat all variables as weak exogenous variables and use their lag values as their instrumental variables. Finally, although the GMM estimator is consistent, it is easy to produce large bias when the sample size is small or the instrumental variables used are weak. Therefore, to ensure the robustness of the test results, a within-fixed-effects estimator will be carried out simultaneously to test the validity of the GMM estimation results.

Supplementary Appendix Table 3 shows the validity test results of the instrumental variables, mainly the autoregressive (AR) and Hansen’s test results, which indicate that the instrumental variables that were adopted are effective. In the regression results, the degree of tax avoidance of peer companies in the current period has a positive impact on the degree of tax avoidance of the enterprises themselves in the current period. That is, listed companies have significant regional peer effects on tax avoidance.

### Heterogeneous Effects

#### The Intensity of Regional Tax Collection and Management

To verify Hypothesis 2a, we drew on Mertens’ (2003) work and used the ratio of the actual tax revenue to the expected tax revenue in each region to measure the intensity of tax collection and administration (*TE*) by local tax authorities. The higher the ratio, the greater the region’s intensity of tax collection and administration. Model (5) introduces the interaction term between the regional peer effects of corporate tax avoidance and the continuous variable tax collection intensity; the regression results are reported in columns 1–4) of Supplementary Appendix Table 4. Simultaneously, to ensure the robustness of the results, the dummy variable, tax collection, and management intensity, is introduced. If the dummy variable is greater than the mean value, the value is 1; otherwise, the value is 0. The regression results are reported in columns 5–8 of Supplementary Appendix Table 4. The regression results show that the coefficient of the cross term is significantly positive; that is, the higher the intensity of tax collection and management in a region, the stronger the regional peer effects of corporate tax avoidance.

#### Informatization Degree of the Region

This paper uses the Internet and telephone penetration rates from the region where the enterprise is located to represent the region’s informatization degree to verify the influence of regional informatization on the regional peer effects of corporate tax avoidance. The higher the Internet and telephone penetration rates in a region, the more diversified the methods of obtaining information within the region and the more convenient the communication between individuals. This paper uses set dummy variables for the Internet penetration rate and telephone penetration rate. When the Internet penetration rate is greater than the mean, the value is 1; otherwise, it is 0. The same is true for telephone penetration. In Model (5), the cross-terms of the core explanatory variable, the dummy variables of Internet penetration and telephone penetration, respectively, were introduced for regression analysis. The regression results are shown in Supplementary Appendix Table 5. The results show that the higher the Internet and telephone penetration rates, the more pronounced the regional peer effects of corporate tax avoidance; this indirectly verifies Hypothesis 2b.

#### Managerial Ownership of the Company

To verify Hypothesis 2c, we defined and measured managerial ownership as the proportion of shares owned by executive directors on the board of directors, consistent with the research of Shan et al. (2019). We introduced dummy variables of the proportion of shares owned by executive directors; when the proportion of shares owned by the executive directors is smaller than the mean value, the value is 1, otherwise, it is 0. The regression results are shown in Supplementary Appendix Table 6. From the regression results, the coefficient of the cross term is significantly positive. That is when managerial ownership is at a level at which the interests of management are discordant with those of the shareholders, the regional peer effect of corporate tax avoidance is more significant.

### Influencing Mechanism Analysis

#### Managers’ Information Learning

To test Hypothesis 3a, we divide the sample into larger and smaller companies and younger and older companies. The regression results are shown in panels A and B in Table 3. The impact of tax avoidance of smaller companies on larger companies and the impact of tax avoidance of younger companies on older companies are not statistically significant. In comparison, the impact of tax avoidance of larger companies on smaller companies and the impact of tax avoidance of older companies on younger companies are statistically significant, at least at the 5% level. The results show that imitating and learning the leading larger and older companies’ behavior produces corporate tax avoidance peer effects.

**TABLE 3:** Managers’ information learning.

	BTD				DR			
	Smaller firms		Larger firms		Smaller firms		Larger firms	
	OLS	2SLS	OLS	2SLS	OLS	2SLS	OLS	2SLS
	(1)	(2)	(3)	(4)	(5)	(6)	(7)	(8)
**Panel A. Size**								
Smaller firms			0.005 (0.007)	0.089 (0.186)			0.016 (0.032)	0.181 (0.163)
Larger firms	0.126*** (0.028)	0.970*** (0.239)			0.066*** (0.017)	0.325** (0.131)		
Control variables	Yes	Yes	Yes	Yes	Yes	Yes	Yes	Yes
Firm\year fixed effects	Yes	Yes	Yes	Yes	Yes	Yes	Yes	Yes
Cons	−0.012 (0.049)		−0.111** (0.049)		−0.028 (0.084)		−0.528*** (0.075)	
*N*	6,773	5,630	4,581	3,737	6,773	5,630	4,581	3,737
*R* ^2^	0.730	–	0.822	–	0.541	–	0.589	–
*K-P F*	–	28.816	–	21.490	–	24.393	–	56.211

	**BTD**				**DR**			
	**Younger firms**		**Older firms**		**Younger firms**		**Older firms**	
	**OLS**	**2SLS**	**OLS**	**2SLS**	**OLS**	**2SLS**	**OLS**	**2SLS**
	**(1)**	**(2)**	**(3)**	**(4)**	**(5)**	**(6)**	**(7)**	**(8)**

**Panel B. Age**								
Younger firms			0.105 (0.088)	0.104 (0.077)			0.187 (0.120)	0.275 (0.179)
Older firms	0.030*** (0.004)	0.196*** (0.059)			0.033*** (0.009)	0.569*** (0.133)		
Control variables	Yes	Yes	Yes	Yes	Yes	Yes	Yes	Yes
Firm\year fixed effects	Yes	Yes	Yes	Yes	Yes	Yes	Yes	Yes
Cons	0.002 (0.008)		−0.330 (0.239)		−0.269*** (0.054)		1.076*** (0.318)	
*N*	5,967	4,555	4,311	3,640	5,967	4,555	4,311	3,640
*R* ^2^	0.973	–	0.850	–	0.633	–	0.619	–
*K-P F*	–	17.042	–	62.297	–	45.671	–	23.829

*Robust standard errors which are clustered at the industry level are presented in parentheses. K-P F value is Kleibergen–Paap F value.*

The symbols *** and ** denote significance at the 0.01 and 0.05 levels, respectively.

#### Manager’ Reputation Consideration

Berkowitz and Kotowitz (1993) believe that the company managers’ reputation incentive has a more significant impact on the initial stages of their careers. Hauser and Warren (1997) believe that an individual’s level of education affects their reputation importance. Therefore, this study draws on the research of Lu et al. (2017) to use three variables: the CEO’s term of office (tenure), age (age), and education level (degree) to measure the degree of the CEO’s attention to reputation. Considering China’s actual corporate governance situation, this paper selects the chairmen of state-owned companies and the general managers of non-state-owned companies as the company’s CEO. We set the dummy variable of the CEO’s term of office and age. When the CEO’s term of office is 30% of the minimum in the region, the value is 1; otherwise, it is 0. When CEO’s age is 30% of the minimum in the region, the value is 1; otherwise, it is 0. We also set the dummy variable of the CEO’s level of education, where 1 means having a graduate degree and above, and 0 means having below a graduate education. Then we introduced the interaction term of explanatory variables and the CEO’s term of office, age, and education level in Model (5). The specific results are shown in Table 4. According to the OLS and 2SLS estimates, the shorter the CEO’s tenure, the younger the age, and the higher the education level, the more significant the peer effects of tax avoidance of listed companies. This indirectly proves Hypothesis 3b.

**TABLE 4:** Managers’ reputation consideration.

	BTD					
	CEO’s age		CEO’s tenure		CEO’s degree	
	OLS	2SLS	OLS	2SLS	OLS	2SLS
	(1)	(2)	(3)	(4)	(5)	(6)
PBTD	0.080* (0.048)	0.983** (0.442)	0.107** (0.053)	0.763*** (0.275)	0.103** (0.048)	0.651* (0.384)
PBTD × age	0.146** (0.065)	1.026** (0.485)				
PBTD × tenure			0.145* (0.087)	1.838** (0.910)		
PBTD × degree					0.102* (0.061)	1.061* (0.543)
Control variables	Yes	Yes	Yes	Yes	Yes	Yes
Firm\year fixed effects	Yes	Yes	Yes	Yes	Yes	Yes
Cons	−0.061 (0.050)		0.030 (0.037)		−0.025 (0.050)	
*N*	7,257	6,321	7,257	6,321	7,257	6,321
*R* ^2^	0.732	–	0.732	–	0.732	–
*K-P F*	–	9.846	–	10.870	–	14.265

	**DR**					
	**CEO’s age**		**CEO’s tenure**		**CEO’s degree**	
	**OLS**	**IV2SLS**	**OLS**	**2SLS**	**OLS**	**2SLS**
	**(1)**	**(2)**	**(3)**	**(4)**	**(5)**	**(6)**

PDR	0.072 (0.090)	1.180* (0.704)	0.326* (0.197)	1.356* (0.704)	0.324* (0.196)	1.367* (0.815)
PDR × age	0.002 (0.003)	1.851* (1.106)				
PDR × tenure			0.290* (0.154)	2.749* (1.653)		
PDR × degree					0.291** (0.130)	1.449* (0.857)
Control variables	Yes	Yes	Yes	Yes	Yes	Yes
Firm\year fixed effects	Yes	Yes	Yes	Yes	Yes	Yes
Cons	−0.260 (0.256)		−0.169 (0.160)		−0.185 (0.227)	
*N*	7,257	6,321	7,257	6,321	7,257	6,321
*R* ^2^	0.499	–	0.500	–	0.501	–
*K-P F*	–	30.373	–	25.363	–	20.352

*Robust standard errors which are clustered at the industry level are presented in parentheses. K-P F value is Kleibergen–Paap F value.*

The symbols ***, **, and * denote significance at the 0.01, 0.05, and 0.1 levels, respectively.

#### Managers’ Information Communication

To verify Hypothesis 3c, we measure the information communication between executives in the same region in two ways. First, when corporate executives have similar individual characteristics, they tend to have similar values and methods; it is easier for them to communicate and learn from each other. Therefore, corporate tax avoidance decisions are more likely to be consistent with peer companies. Second, in China’s social culture, it is easier for senior executives from the same native place to communicate and learn through “fellow villagers’ meetings” and other forms. Therefore, this paper draws on the research of Parsons et al. (2018) and uses executives’ native place, age, and educational background as indirect proxy variables for communication between executives.

First, this study selected a sample of at least two executives from listed companies in the same region who have the same hometown to analyze the impact of peer companies with the “Hometown” relationship on corporate tax avoidance decision-making. Second, we divided the samples into younger executives (less than or equal to the average age) and older executives (greater than the average age), higher education (graduate and above), and lower education (below graduate education) for analysis. The regression results are shown in Table 5. According to the OLS and 2SLS estimates, the coefficient of tax avoidance behavior for the company with a “Hometown” relationship on the tax avoidance decisions in panel A is significantly greater than that in the full sample regression (0.076 and 0.143, respectively). This shows that the social network communication behavior based on a “Hometown” relationship will impact peer effects among industries. In panels B and C, the peer effects for listed companies of a similar age and educational background are more significant. When the individual characteristics of executives are more similar, it is easier for executives to communicate and learn from each other. Hence, the peer effects of tax avoidance are more significant.

**TABLE 5:** Managers’ information communication.

	BTD		DR	
	OLS	2SLS	OLS	2SLS
	(1)	(2)	(3)	(4)
**Panel A. “Hometown” relationship**				
PBTD	0.232** (0.097)	3.205** (1.481)		
PDR			0.390** (0.186)	1.589* (0.948)
Control variables	Yes	Yes	Yes	Yes
Firm\year fixed effects	Yes	Yes	Yes	Yes
Cons	0.127 (0.455)		−1.338*** (0.509)	
*N*	442	420	442	420
*R* ^2^	0.802	–	0.622	–
*K-P F*	–	17.494	–	16.860

	BTD				DR			
	Younger executives		Older executives		Younger executives		Older executives	
	OLS	2SLS	OLS	2SLS	OLS	2SLS	OLS	2SLS
	(1)	(2)	(3)	(4)	(5)	(6)	(7)	(8)
**Panel B. Age of the executive**								
Younger executives	0.838*** (0.010)	1.054*** (0.084)			0.924*** (0.006)	1.031*** (0.038)		
Older executives			0.841*** (0.013)	1.325*** (0.129)			0.907*** (0.012)	1.256*** (0.159)
Control variables	Yes	Yes	Yes	Yes	Yes	Yes	Yes	Yes
Firm\year fixed effects	Yes	Yes	Yes	Yes	Yes	Yes	Yes	Yes
Cons	0.063*** (0.010)		−0.013 (0.012)		0.063*** (0.021)		−0.005 (0.029)	
*N*	5,576	4,364	4,536	3,872	5,576	4,364	4,536	3,872
*R* ^2^	0.932	–	0.908	–	0.960	–	0.961	–
*K-P F*	–	31.620	–	59.865	–	61.469	–	49.479

	**BTD**				**DR**			
	**Higher education**		**Lower education**		**Higher education**		**Lower education**	
	**OLS**	**2SLS**	**OLS**	**2SLS**	**OLS**	**2SLS**	**OLS**	**2SLS**
	**(1)**	**(2)**	**(3)**	**(4)**	**(5)**	**(6)**	**(7)**	**(8)**

**Panel C. Educational background of the executive**								
Higher education	0.915*** (0.021)	0.986*** (0.055)			0.937*** (0.012)	1.107*** (0.036)		
Lower education			0.841*** (0.009)	0.965*** (0.058)			0.900*** (0.010)	0.991*** (0.136)
Control variables	Yes	Yes	Yes	Yes	Yes	Yes	Yes	Yes
Firm\year fixed effects	Yes	Yes	Yes	Yes	Yes	Yes	Yes	Yes
Cons	−0.033* (0.019)		0.044*** (0.009)		0.040 (0.026)		−0.034 (0.021)	
*N*	2,092	1,461	3,839	3,003	2,092	1,461	3,839	3,003
*R* ^2^	0.941	–	0.953	–	0.973	–	0.936	–
*K-P F*	–	59.084	–	52.589	–	76.871	–	39.836

*Robust standard errors which are clustered at the industry level are presented in parentheses. K-P F value is Kleibergen–Paap F value.*

The symbols ***, **, and * denote significance at the 0.01, 0.05, and 0.1 levels, respectively.

## Conclusion

This study investigates if there are significant regional peer effects in tax avoidance. In the analysis of heterogeneity, we found that for companies with a stronger intensity of regional tax collection and management, a higher degree of informatization, and companies with a low management shareholding ratio, the regional peer effects of enterprise tax avoidance are more significant. Finally, we found that managers’ information learning, reputation concern, and information communication influence peer effects.

Based on the aforementioned conclusions, we noted the following policy implications. First, there are significant regional peer effects in corporate tax avoidance decision-making, indicating that corporate tax avoidance behavior has a significant “contagion” among geographically close enterprises. The tax avoidance behavior of peer enterprises sends signals to other enterprises in the same region and creates a certain incentive effect. This will not only enhance the tax avoidance motivation of other enterprises but also make those enterprises without tax avoidance motivation imitate the tax avoidance. Therefore, tax authorities should pay attention to the existence of corporate tax avoidance peer effects to prevent or avoid the “contagion” of tax avoidance. Second, the peer effects of corporate tax avoidance behavior have noticeable differences between regions and enterprises. Therefore, tax departments should consider these differences, explore and summarize the tax avoidance rules of different regions and different types of enterprises, establish a tax risk identification analysis for similar companies, and increase the probability of tax authorities inspecting and auditing key enterprises and regions of tax avoidance. Third, as the information communication between and the reputations of corporate executives will encourage enterprises to imitate the tax avoidance decisions of the peer enterprises, the government should actively strengthen corporate executives’ guidance and management. Hence, it is necessary to standardize the public disclosure and dissemination of listed companies’ information for avoiding unreasonable tax avoidance information transmission among enterprises. Simultaneously, tax payments’ publicity in accordance with the law should be strengthened. Senior executives of enterprises with a high degree of tax compliance should be regularly selected to teach compliance practices and share relevant experiences to other senior executives as well as actively transmit signals of tax payment in accordance with the law to the peer enterprises for a positive peer effect. Further, we should give importance to the supervisory role of independent directors, and the boards of supervisors, and investors; improve the channels and ways for investors to participate in corporate governance; and optimize the governance of the board of directors to better monitor and restrain the managers’ opportunistic behaviors. Appropriate incentive measures should be adopted to eliminate the cognitive bias of senior executives and reduce or eliminate the tax avoidance behavior of imitating peer enterprises due to senior executives’ motive for self-interest.

## Data Availability Statement

The raw data supporting the conclusions of this article will be made available by the authors, without undue reservation.

## Author Contributions

YG: conceptualization, methodology, software, validation, formal analysis, data curation, writing – original draft preparation, writing – review and editing, and visualization. YC: resources and investigation. CC: supervision. All authors have read and agreed to the published version of the manuscript.

## Conflict of Interest

The authors declare that the research was conducted in the absence of any commercial or financial relationships that could be construed as a potential conflict of interest.

## Publisher’s Note

All claims expressed in this article are solely those of the authors and do not necessarily represent those of their affiliated organizations, or those of the publisher, the editors and the reviewers. Any product that may be evaluated in this article, or claim that may be made by its manufacturer, is not guaranteed or endorsed by the publisher.

## Footnotes

1 An enterprise’s cash income tax rate is equal to its actual income tax cash flow expenditure/total profit. Refer to Li et al. (2017) for the actual income tax cash flow expenditure of an enterprise = the income tax expense of the enterprise − deferred income tax expense + income tax payable at the beginning − income tax payable at the end of the period. Therefore, cash flow positively correlates with corporate tax avoidance. Further, the higher the idiosyncratic return rate of a company’s stock, the more expected cash flow the company will have, which indicates that corporate tax avoidance is positively correlated with the idiosyncratic return rate of a company’s stock.

2 The specific calculation method of the formula is as follows: $d=2r^{*}arcsin\left(\sqrt{sin^{2}\left(\frac{\theta_{2}-\theta_{1}}{2}\right)+cos\left(\theta_{1}\right)cos\left(\theta_{2}\right)sin^{2}\left(\frac{\theta_{2}-\theta_{1}}{2}\right)}\right)$, where *d* represents the distance between two enterprises in kilometers; θ_2_ and θ_1_ are the latitudes of the first and second enterprises; and ψ_2_ and ψ_1_ are the longitudes of the first and second enterprises. *r* is the radius of the Earth, 6,378.137 km.

3 The calculation method is as follows: $PeerTax_{-i}^{c}=\left(\sum Tax_{-i}^{c}-\sum Tax_{i}^{c}\right)/\left(N^{c}-1\right)$, where *c* represents the circle centered on company *i*, $Tax_{-i}^{c}$ represents the tax avoidance degree of company *i* in circle *c*, and *N*^*c*^ represents the number of all listed companies within the range of circle *c*.
