# Supplementary material for: Regional Peer Effects of Corporate Tax Avoidance
Source: Front Psychol. 2021 Dec 7;12:744371. doi: 10.3389/fpsyg.2021.744371 (PMC8688992; doi:10.3389/fpsyg.2021.744371)
Supplement: Supplementary file 1 [file Data_Sheet_1.pdf]

Online appendix

Table 1. Robustness checks—Adjusting the measurement method of tax avoidance degree

	ETR1		ETR2	
	OLS (1)	2SLS (2)	OLS (3)	2SLS (4)
PETR1	0.016** (0.008)	0.151** (0.067)		
PETR2			0.009* (0.005)	0.103* (0.061)
Control variables	Yes	Yes	Yes	Yes
Firm\Year fixed effects	Yes	Yes	Yes	Yes
cons	0.829 (0.816)		0.181 (0.275)	
N	12,328	11,409	12,328	11,409
R^2	0.667	-	0.575	-
K-P F	-	57.905	-	89.403

Notes: (1) Robust standard errors clustered at the industry level are presented in parentheses. (2) ***, **, and * denote significance at the 0.01, 0.05, and 0.1 levels, respectively. (3) K-P F value is Kleibergen-Paap F value.

Table 2. Robustness checks—Change the identification method of peer companies

	BTD				*DR*			
	175KM (1)	200KM (2)	225KM (3)	250KM (4)	175KM (5)	200KM (6)	225KM (7)	250KM (8)
PBTD	0.014*** (0.004)	0.010** (0.004)	0.009** (0.004)	0.008 (0.010)				
PDR					0.007** (0.003)	0.007** (0.003)	0.005 (0.003)	0.004 (0.003)
Control variables	Yes	Yes	Yes	Yes	Yes	Yes	Yes	Yes
Firm\Year fixed effects	Yes	Yes	Yes	Yes	Yes	Yes	Yes	Yes
cons	-0.051*** (0.013)	-0.051*** (0.013)	-0.051* (0.030)	-0.053* (0.030)	-0.060** (0.025)	-0.062** (0.025)	-0.062** (0.025)	-0.062** (0.025)
N	12,328	12,328	12,328	12,328	12,328	12,328	12,328	12,328
R^2	0.140	0.141	0.141	0.140	0.118	0.118	0.118	0.117

Notes: (1) Robust standard errors clustered at the industry level are presented in parentheses. (2) ***, **, and * denote significance at the 0.01, 0.05, and 0.1 levels, respectively.

Table 3. Robustness checks—Dynamic data model

	GMM	GMM
	BTD (1)	DR (2)
L. BTD	0.680*** (0.005)	
L. DR		0.342*** (0.004)
PBTD	0.049*** (0.011)	
PDR		0.286*** (0.017)
SIZE	0.014*** (0.001)	0.031*** (0.002)
ROA	0.085*** (0.004)	0.565*** (0.014)
LEV	-0.028*** (0.002)	-0.096*** (0.005)
ROI	0.274*** (0.010)	0.487*** (0.023)
	-0.022***	-0.028***

PPE	(0.002)	(0.006)
INVENT	-0.013*** (0.002)	-0.025*** (0.007)
INTANG	-0.032*** (0.006)	-0.075*** (0.015)
AGE	0.006 (0.010)	-0.034 (0.024)
PSIZE	-0.000 (0.001)	0.001 (0.002)
PROA	-0.050*** (0.010)	-0.074** (0.029)
PLEV	-0.022*** (0.002)	0.028*** (0.008)
PROI	0.377*** (0.035)	0.150 (0.096)
PPPE	-0.013*** (0.004)	-0.064*** (0.013)
PINVENT	0.028*** (0.006)	-0.069*** (0.019)
PINTANG	-0.016 (0.013)	0.103** (0.040)
PAGE	-0.023*** (0.002)	-0.024*** (0.008)
cons	0.005 (0.011)	0.006 (0.042)
Abond test for AR (1)	[0.000]	[0.000]
Abond test for AR (2)	[0.654]	[0.362]
Hansen test	[0.188]	[0.305]
N	9,080	9,080

Notes: (1) Robust standard errors clustered at the industry level are presented in parentheses. (2) ***, **, and * denote significance at the 0.01, 0.05, and 0.1 levels, respectively. (3) The value in [] represents the p-value of the corresponding statistic.

Table 4. Heterogeneous Effects—The intensity of regional tax collection and management

	BTD		*DR*		*BTD*		*DR*	
	OLS (1)	2SLS (2)	OLS (3)	2SLS (4)	OLS (5)	2SLS (6)	OLS (7)	2SLS (8)
PBTD	0.001 (0.058)	0.229 (0.697)			0.059* (0.036)	0.528* (1.423)		
PDR			0.181*** (0.061)	0.303 (1.946)			0.124* (0.072)	0.612 (0.492)
*PBTD *TE*	0.087 (0.076)	0.705** (0.330)						
*PDR *TE*			0.111** (0.057)	0.960 (1.989)				
*PBTD *dum_TE*					0.124* (0.074)	1.066*** (0.381)		
*PDR *dum_TE*							0.157* (0.090)	1.042* (0.622)
Control variables	Yes	Yes	Yes	Yes	Yes	Yes	Yes	Yes
Firm\Year fixed effects	Yes	Yes	Yes	Yes	Yes	Yes	Yes	Yes
cons	-0.081 (0.072)		0.169 (0.204)		-0.043 (0.051)		-0.091 (0.050)	
N	12,328	11,409	12,328	11,409	12,328	11,409	12,328	11,409
R^2	0.726	-	0.874	-	0.726	-	0.874	-
K-P F	-	20.973	-	39.989	-	26.577	-	41.614

Notes: (1) Robust standard errors clustered at the industry level are presented in parentheses. (2) ***, **, and * denote significance at the 0.01, 0.05, and 0.1 levels, respectively. (3) K-P F value is Kleibergen-Paap F value.

Table 5. Heterogeneous Effects—Informatization degree of the region

	internet		*phone*	
	BTD	*DR*	*BTD*	*DR*

	OLS (1)	2SLS (2)	OLS (3)	2SLS (4)	OLS (5)	2SLS (6)	OLS (7)	2SLS (8)
PBTD	0.118*** (0.043)	0.225* (0.133)			0.113** (0.043)	0.391** (0.196)		
PDR			0.114* (0.067)	1.167* (0.704)			0.126* (0.066)	0.366 (4.088)
*PBTD *internet*	0.116* (0.070)	0.568** (0.242)						
*PDR *internet*			0.231* (0.135)	1.156* (0.633)				
*PBTD *phone*					0.152** (0.066)	0.920* (0.489)		
*PDR *phone*							0.257* (0.155)	1.234* (0.707)
Control variables	Yes	Yes	Yes	Yes	Yes	Yes	Yes	Yes
Firm\Year fixed effects	Yes	Yes	Yes	Yes	Yes	Yes	Yes	Yes
cons	-0.161 (0.107)		0.063 (0.197)		-0.245** (0.120)		0.254 (0.175)	
N	10,776	9,737	10,776	9,737	10,791	9,750	10,791	9,750
R^2	0.944	-	0.517	-	0.820	-	0.524	-
K-P F	-	49.191	-	50.636	-	78.419	-	16.259

Notes: (1) Robust standard errors clustered at the industry level are presented in parentheses. (2) ***, **, and * denote significance at the 0.01, 0.05, and 0.1 levels, respectively. (3) K-P F value is Kleibergen-Paap F value.

Table 6. Heterogeneous Effects—Managerial ownership of the company

	BTD		DR	
	OLS (1)	2SLS (2)	OLS (3)	2SLS (4)
PBTD	0.560** (0.249)	0.229 (0.697)		
PDR			0.002 (0.019)	0.101** (0.050)
PBTD *dum_MOS	0.449*** (0.172)	4.252** (1.770)		
PDR *dum_MOS			0.021 (0.019)	2.863*** (0.973)
Control variables	Yes	Yes	Yes	Yes
Firm\Year fixed effects	Yes	Yes	Yes	Yes
cons	0.541*** (0.181)		1.777*** (0.550)	
N	1,701	1,649	1,701	1,649
R^2	0.921	-	0.715	-
K-P F	-	15.052	-	25.550

Notes: (1) Robust standard errors clustered at the industry level are presented in parentheses. (2) ***, **, and * denote significance at the 0.01, 0.05, and 0.1 levels, respectively. (3) K-P F value is Kleibergen-Paap F value.
